# Identification and validation of a five-gene prognostic signature for hepatocellular carcinoma

**DOI:** 10.1186/s12957-021-02202-9

**Published:** 2021-03-26

**Authors:** Huibin Yang, Junyu Huo, Xin Li

**Affiliations:** 1 Qingdao University, No. 308 Ningxia Road, Qingdao, 266071 China; 2 Department of Hepatobiliary and Pancreatic Surgery, The Affiliated Qingdao Municipal Hospital of Qingdao University, No.1 Jiaozhou Road, Shibei District, Qingdao City, 266011 Shandong Province China

**Keywords:** Hepatocellular carcinoma, ARID1A mutations, Prognostic, Signature

## Abstract

**Background:**

ARID1A is a commonly mutated tumor suppressor gene found in all human cancer types, but its clinical significance, oncogenic functions, and relevant mechanisms in hepatocellular carcinoma (HCC) are not well understood.

**Objective:**

We aimed to improving the prognosis risk classification of HCC from the perspective of ARID1A mutations.

**Materials and methods:**

We examined the interaction between ARID1A mutations and the overall survival via Kaplan-Meier survival analysis. We used gene set enrichment analysis (GSEA) to elucidate the influence of ARID1A mutations on signaling pathways. A prognostic model was constructed using LASSO and multivariate Cox regression analyses. A receiver operating characteristic (ROC) curve was used to estimate the performance and accuracy of the model.

**Results:**

HCC patients with ARID1A mutations presented poor prognosis. By GSEA, we showed that genes upregulated by reactive oxygen species (ROS) and regulated by MYC were positively correlated with ARID1A mutations. A prognostic signature consisting of 5 genes (SRXN1, LDHA, TFDP1, PPM1G, and EIF2S1) was constructed in our research. The signature showed good performance in predicting overall survival (OS) for HCC patients by internal and external validation.

**Conclusion:**

Our research proposed a novel and robust approach for the prognostic risk classification of HCC patients, and this approach may provide new insights to improve the treatment strategy of HCC.

**Supplementary Information:**

The online version contains supplementary material available at 10.1186/s12957-021-02202-9.

## Background

A recent study on the next-generation sequencing of 669 patients with 24 types of cancers showed that the average mutation rate of the SWI/SNF gene was 19%, which was only lower than the mutation rate of TP53 (26%); therefore, the SWI/SNF gene is ranked second among all mutated genes, making the SWI/SNF complex and its mediated nucleosome chromatin remodeling the focus of cancer research [1]. The ARID1A (AT-rich binding domain 1A) gene, also known as BAF250a, is the core subunit of the chromatin remodeling complex SWI/SNF [2, 3] and may help to control the specificity exhibited by SWI/SNF in the whole genome nucleosome remodeling and affect the composition and catalytic ability of the whole complex [4]. The chromatin remodeling complex participates in the process of DNA replication, transcription, and repair, participates in the regulation of the PI3K/Akt signaling pathway, and carries out chromatin remodeling with the histone covalent modification complex, which is related to the inhibition of tumorigenesis and development [5, 6].

Recently, research on the function and mechanism of ARID1A in tumors have made important progress. ARID1A inhibits cell proliferation by regulating the cell cycle, induces the expression of P21 factor, promotes its binding to the cyclin CDK2/CDK4 complex, and inhibits its activity so that the cell cycle is stagnated in the G1 phase. ARID1A expression is downregulated in other stages, except in the G0 phase, and is almost completely deficient in cells with exuberant cell division [3]. ARID1A deficiency can change the gene expression profile of embryonic stem cells, increase the expression of the cell development-related genes Gata4, Ga-ta6, Tnt2, and Myl3, and decrease the expression of stem cell self-renewal genes, which further confirms the importance of ARID1A in stem cell maintenance and differentiation [6]. The ARID1A gene was knocked out in leukemic cells, and it was found that apoptosis mediated by fas was inhibited [7]. ARID1A can also promote apoptosis by regulating the target genes Bcl-2 and cyclin D1 [7]. Therefore, ARID1A, as an important tumor suppressor gene, may remarkably affect tumor occurrence and development.

Huang et al. [8] used exon sequencing technology to detect somatic mutations in 110 patients with portal vein tumor thrombi (PVTTs) and patients with HBV-positive hepatocellular carcinoma (HCC). They found that the mutation of the ARID1A gene was the most important, and the mutation rate of the ARID1A gene was 13% (14/110), indicating that it remarkably affected HCC occurrence and development. Increasing evidences suggested that decreased expression of ARID1A in HCC patients was associated with poor prognosis and could promoted metastasis of HCC [9, 10], and ARID1A could also regulate response to anti-angiogenic therapy in advanced HCC [11], implying that ARID1A may represent a promising candidate therapeutic target for HCC. However, the molecular mechanism of ARID1A in HCC remains to be clarified.

The study exploring the potential molecular mechanism for ARID1A mutation with HCC via gene set enrichment analysis (GSEA). We proposed a five-gene signature to evaluate HCC prognosis and carried out internal and external verification, which will guide the clinical management of HCC.

## Materials and methods

### Data collection

We downloaded the mRNA expression profile of 374 HCC samples from The Cancer Genome Atlas (TCGA-LIHC) website (https://portal.gdc.cancer.gov/projects/TCGA-LIHC). Corresponding clinical information was available on the UCSC Xena website (https://tcga-xena-hub.s3.us-east-1.amazonaws.com/latest/TCGA.LIHC.sampleMap%2FLIHC_clinicalMatrix; Full metadata). We acquired the sample list of ARID1A alterations from the cBioPortal website (https://www.cbioportal.org/). We obtained sequence data, somatic mutation data, together with the corresponding clinical data for 228 HCC samples from the International Cancer Genomics Consortium (ICGC-LIRI-JP, https://dcc.icgc.org/projects/LIRI-JP). TCGA and ICGC were all based on Illumina HiSeq platform. This study meets the publication requirements of the TCGA and ICGC. The detailed clinical information of all included samples as shown in Table 1. The data of this study were from a public database, so they do not need to be approved by the local ethics committee.

**Table 1:** The detailed clinical information of all included samples

	TCGA	ICGC
Survival status		
Alive	224	187
Dead	119	41
Age		
≤ 65	127	88
> 65	216	140
Gender		
Male	233	167
Female	110	61
Stage		
I–II	238	140
III–IV	83	88
Prior malignancy		
Yes	31	29
No	312	199
Grade		
G1	53	
G2	161	
G3–4	124	
BMI		
≤ 25	163	
> 25	153	
AFP		
≤ 300 ng/ml	197	
> 300 ng/ml	62	
New tumor event after initiate treatment		
Yes	168	
No	162	
Cancer status		
With tumor	149	
Tumor free	179	
Vascular tumor cell type		
None	188	
Micro and marco	101	
Race		
White	169	
Others	164	
Additional_pharmaceutical_therapy		
No	108	
Ye	29	
Residual_tumor		
R0	303	
R1–2	16	
ARID1A		
Mutation	25	23
Wild	306	203

### Gene set enrichment analysis (GSEA)

To explore the underlying molecular mechanism regarding ARID1A mutations in HCC, we performed gene set enrichment analysis on the samples with (*n* = 36) and without (*n* = 338) ARID1A mutation. To identify the pathways that were significantly enriched between patients with and without ARID1A mutations, we selected an annotated gene set file (h.all.v7.1.symbols.gmt) as the reference. The threshold was confirmed as NOM *p* value < 0.05; FDR *q* value < 0.25. GSEA software (http://www.broadinstitute.org/gsea) was applied to GSEA.

### Construction of a five-gene prognostic signature

The “caret” package in R software assisted in classifying the 343 HCC patients who survived more than one month from the TCGA-LIHC project into the training cohort (*n* = 172) and the testing cohort (*n* = 171) of equal sample size in a random manner. No statistically significant difference in the clinico-pathological parameter was detected between the training and validation set by the chi-square test (supplement material 2), which meaned means that we excluded the effect of clinical factors on the prognosis of patients in both cohorts. We extracted gene sets significantly upregulated in ARID1A mutation samples, and univariate Cox regression analysis assisted in identifying prognostic genes in the training cohort. Then, important mRNAs were confirmed from the prognostic genes via the least absolute shrinkage and selection operator (LASSO) model. Multivariate Cox proportional regression analysis served to establish a risk score model with the identified mRNAs. The Kaplan-Meier (KM) analysis together with the receiver operating characteristic (ROC) curve assisted in evaluating the clinical value exhibited by the risk score. Univariate and multivariate Cox regression helped analyze the factors related to prognosis in HCC patients.

### The five-gene prognostic signature validation

After the construction of the prognostic signature, we used the testing cohort (*n* = 171) together with the entire TCGA cohort (*n* = 343) to verify the accuracy exhibited by the prognostic risk model. To ensure the stability of the validation results, patients from TCGA were grouped according to their clinical features, and survival analysis was carried out by subgroup. An independent dataset (ICGC, *n* = 228) was employed for external validation for its effectiveness in overall survival (OS) prediction specific to HCC patients.

## Results

### ARID1A alteration associated with increased gene mutation count

**Fig. 1:**
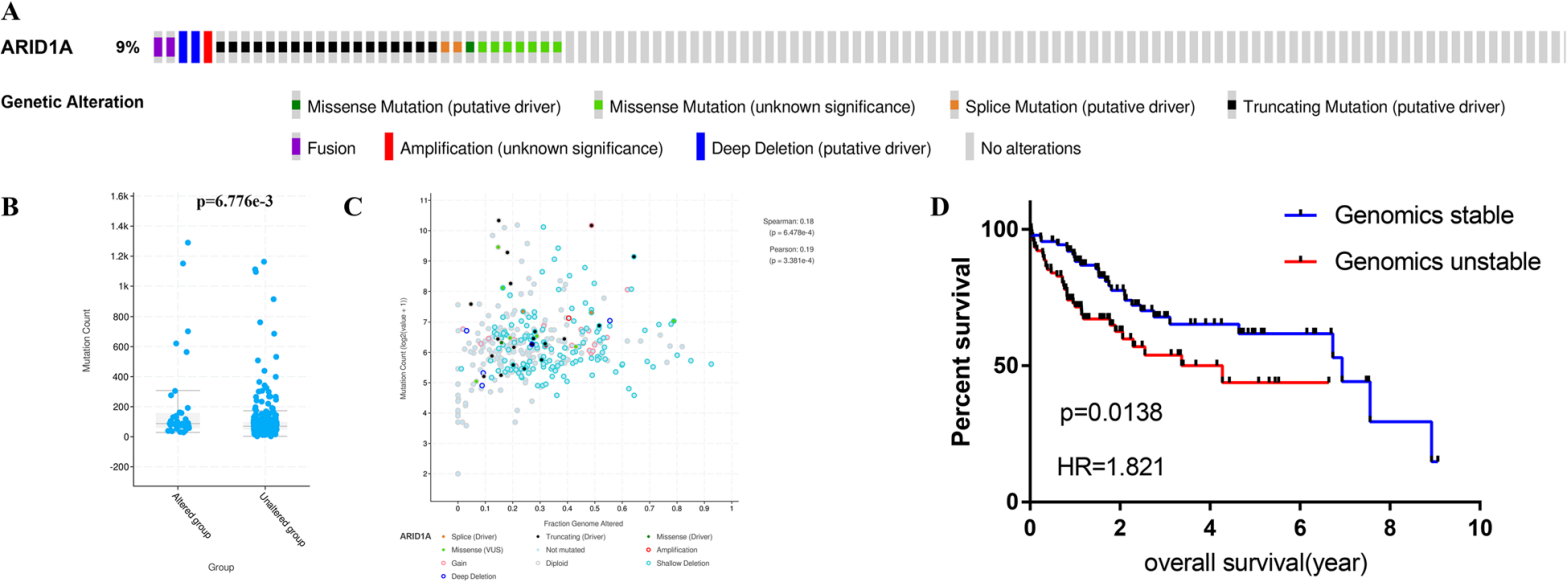
The landscape of ARID1A alteration in TCGA. **a** The mutation type of ARID1A. **b**, **c** The relationship between the fraction of ARID1A-altered with gene mutation count. **d** The Kaplan-Meier survival analysis regarding genomic unstable (GU) group and genomic stable (GS) group

The mutation frequency of ARID1A in TCGA datasets was 9%. Truncating mutation was the main mutation type, followed by missense mutation, and amplification was the rarest (Fig. 1a). The gene mutation count of the ARID1A-altered group was higher than that in the unaltered group, which was statistically significant (Fig. 1b). The fraction of ARID1A-altered exhibited positive correlation with gene mutation count (Fig. 1c). Interestingly, the top 25% of samples with the highest gene mutation count were assigned into the genomic unstable (GU) group, whereas the bottom 25% with the lowest gene mutation count were classified as genomic stable (GS) group [12], the two groups differed greatly in overall survival (OS) (Fig. 1d). The somatic mutation data (TCGA.LIHC.varscan.somatic.maf) were downloaded from TCGA (https://tcga-data.nci.nih.gov/tcga/). The subsequent workflow chart of this research is shown in supplement file 1.

### Identification of gene sets enriched in ARID1A-mutated HCC samples

**Fig. 2:**
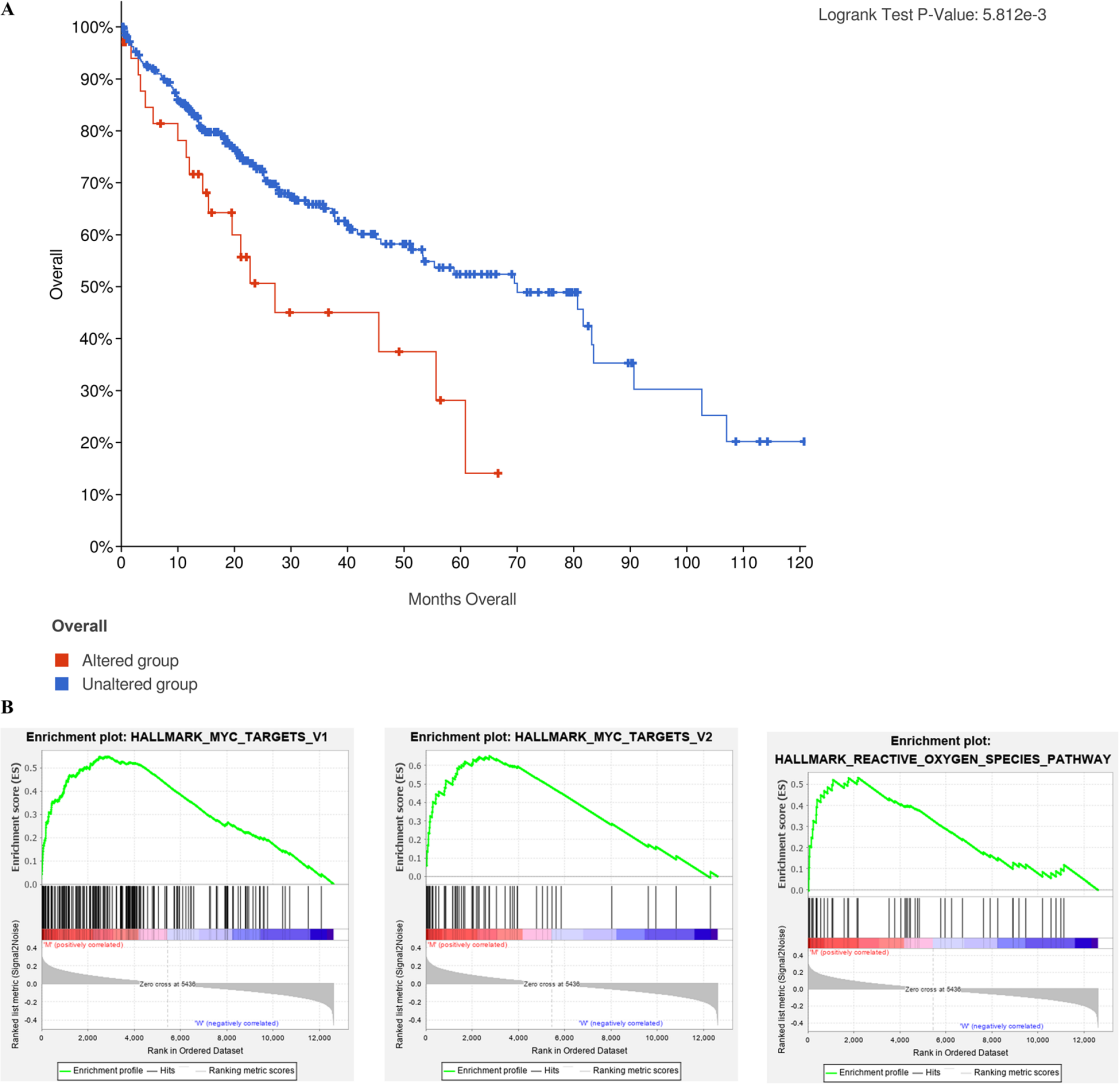
The Kaplan-Meier survival analysis of ARID1A alteration for HCC. **a** The Kaplan-Meier survival curve. **b** Gene sets are upregulated in phenotype ARID1A alteration (FDR < 0.25 and nominal *p* value < 0.05)

By comparing the OS of HCC patients with ARID1A mutations and those without ARID1A mutations, we found that the OS of those with ARID1A mutations was obviously lower than that of those without ARID1A mutations (Fig. 2a). We conducted GSEA on the altered group and unaltered group to investigate the underlying molecular mechanism. After screening, three gene sets were considered to be positively correlated with ARID1A mutations (Fig. 2b, Table 2). A total of 288 genes were extracted for the subsequent analyses.

**Table 2:** Gene set enrichment analysis for ARID1A alteration HCC samples

Gene sets	ES	NES	NOM p-val	FDR q-val
HALLMARK_MYC_TARGETS_V2	0.650381	1.851576	0.015521	0.19292
HALLMARK_REACTIVE_OXYGEN_SPECIES_PATHWAY	0.53105	1.770816	0.024176	0.187634
HALLMARK_MYC_TARGETS_V1	0.549157	1.754343	0.036403	0.14646

### Construction of a five-gene prognostic signature based on the training cohort

**Fig. 3:**
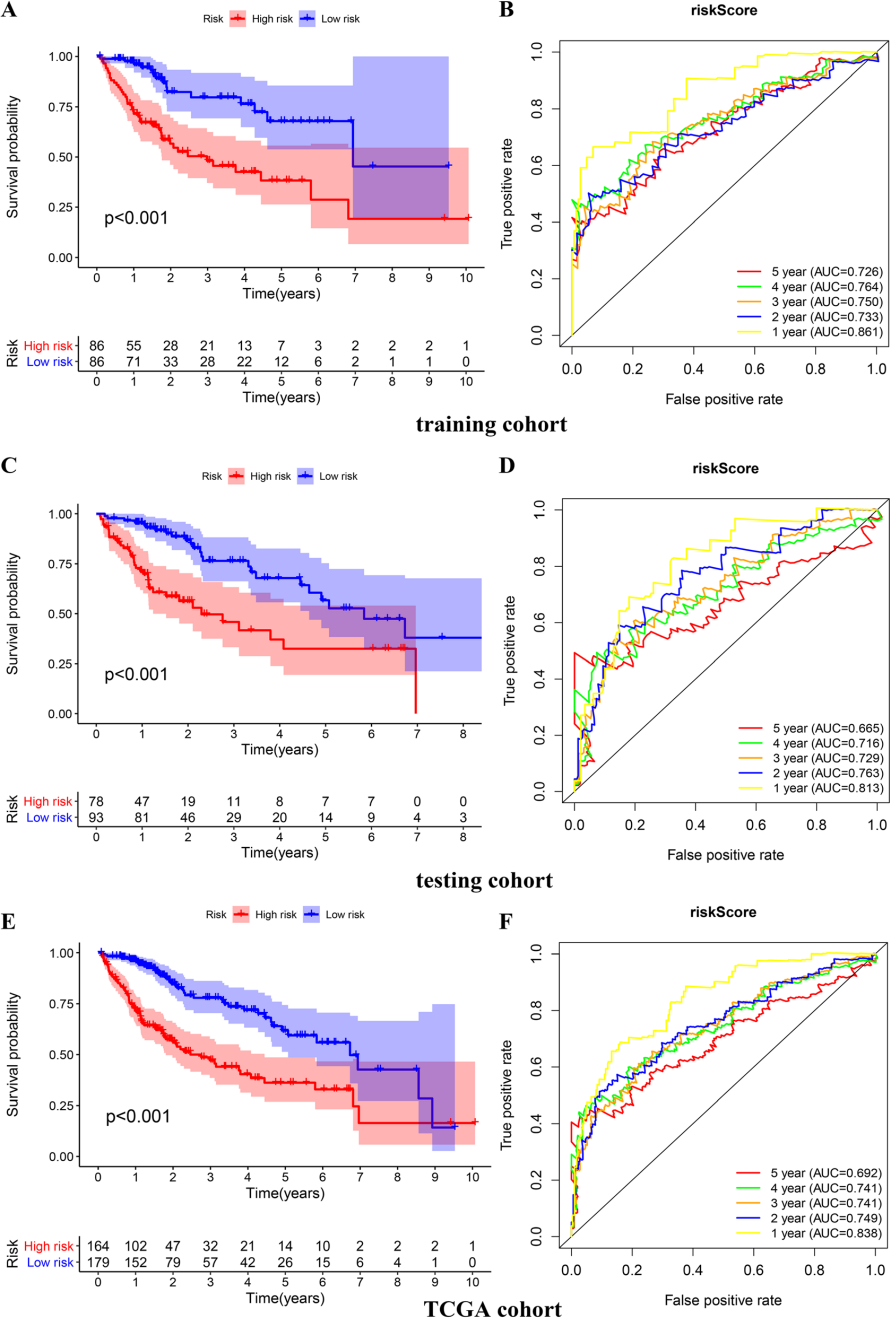
The establishment of the 5-gene signature. **a** Kaplan-Meier survival analysis and time-dependent ROC analysis of predicting overall survival for patients in training cohort used by risk score. **b** Kaplan-Meier survival analysis and time-dependent ROC analysis of predicting overall survival for patients in testing cohort used by risk score. **c** Kaplan-Meier survival analysis and time-dependent ROC analysis of predicting overall survival for patients in whole TCGA cohort used by risk score

As revealed by univariate Cox regression analysis, 28 genes exhibited an association with patient OS (*p* < 0.001, Table 3). Then, genes that may show a close relation to other genes were excluded through LASSO regression analysis. The 8 genes selected from LASSO regression analysis were retained for multivariate Cox regression analysis. These prognostic gene expression markers were linearly combined with the regression coefficient (*β*) in the multivariate Cox regression analysis to construct a risk score model. The risk score was calculated as follows: SRXN1*0.03974+ LDHA*0.003247+ TFDP1*0.022758+ PPM1G*0.037018+ EIF2S1*0.063182. The median risk score (0.85) was taken as the threshold for dividing patients in the training set into a group with a high risk and a group with a low risk. As shown in the KM survival curve, the prognosis of patients in the group with a high risk presented statistical significance (Fig. 3a). The area under the ROC curve (AUC) values for the risk score in predicting 1-year, 2-year, 3-year, 4-year, and 5-year OS were 0.861, 0.733, 0.750, 0.764, and 0.726, respectively (Fig. 3b), indicating that this prognostic signature has a better predictive power for HCC patient survival.

**Table 3:** The prognostic gene list

Gene name	HR	HR.95L	HR.95H	*p* value
TXNRD1	1.014623	1.008958	1.020319	3.73E–07
TFDP1	1.03243	1.019555	1.045468	6.21E–07
SRXN1	1.048058	1.028743	1.067735	7.58E–07
EIF2S1	1.222953	1.125922	1.328346	1.82E–06
PSMD1	1.096507	1.055521	1.139086	2.14E–06
PPM1G	1.049258	1.027294	1.071692	8.40E–06
GCLM	1.031107	1.017293	1.045107	8.53E–06
KPNA2	1.01951	1.010513	1.028586	1.93E–05
LDHA	1.005669	1.003064	1.008281	1.94E–05
MCM6	1.06796	1.036161	1.100735	2.02E–05
SERBP1	1.050061	1.026388	1.074281	2.69E–05
RRM1	1.056424	1.029482	1.084072	3.12E–05
PRDX1	1.002777	1.001431	1.004125	5.23E–05
PSMB2	1.041963	1.021234	1.063114	6.09E–05
CCT4	1.028998	1.014373	1.043834	9.08E–05
MCM2	1.060914	1.029698	1.093077	0.000104
USP1	1.096038	1.045936	1.148539	0.000122
CCT5	1.024885	1.011956	1.03798	0.000148
GSR	1.022544	1.010775	1.034451	0.00016
ETF1	1.070413	1.033074	1.109102	0.000173
XPO1	1.061489	1.027121	1.097007	0.00038
PSMD14	1.060731	1.026673	1.095918	0.000399
CAD	1.124055	1.053586	1.199237	0.0004
PSMA1	1.029786	1.013009	1.046842	0.000462
CUL1	1.070192	1.029964	1.111991	0.00052
NCBP1	1.234655	1.094769	1.392415	0.000591
CCT7	1.017927	1.007499	1.028462	0.00072
CCT3	1.007581	1.003075	1.012108	0.000957

### Internal validation of the five-gene prognostic signature in the testing cohort

The risk score regarding the 171 patients in the testing set was calculated further. We divided them into a group with a high risk and a group with a low risk considering the same cutoff point (0.85) in the training set. As shown in Fig. 3c, patients in the high-risk group presented an obviously lower OS than those in the low-risk group. The risk score provided the highest 1-year AUC (0.813), which was still considered to represent a high predictive efficacy (Fig. 3d).

### Validation of the five-gene prognostic signature in the TCGA cohort

To validate the accuracy exhibited by the risk model, we analyzed the model in the entire TCGA cohort. Consistent with the foregoing results, patients in the high-risk group had a substantially worse outcome than patients in the low-risk group (*P* < 0.001) (Fig. 3e). The AUC ranged from 0.692 to 0.838, which showed an excellent predictive capability of the model (Fig. 3f).

### Subgroup analysis of the five-gene prognostic signature

**Fig. 4:**
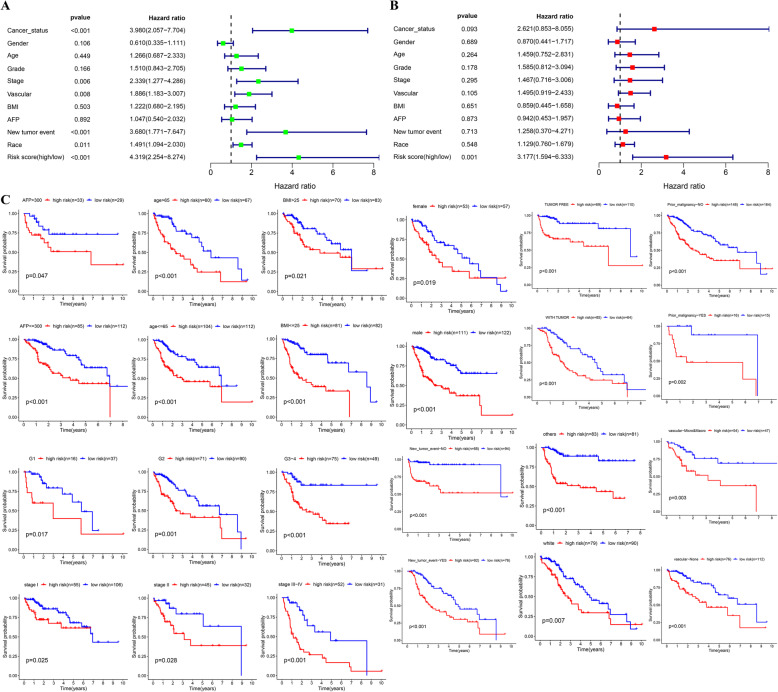
Independence validation of the risk score for predicting overall survival of HCC in the TCGA cohort. **a** Univariate Cox analysis. **b** Multivariate Cox analysis. **c** Subgroup survival analysis based on clinical features

From the results of both univariate and multivariate Cox regression analyses, the risk score was found to be an independent prognostic indicator (Fig. 4a, b), and we validated the applicability of the prognostic value given by our model to other clinical factors. We divided the patients into 24 subgroups based on their clinicopathologic characteristics, and next, each subgroup was further separated into a group with a high risk and a group with a low risk relying on the 5-gene signature. Kaplan-Meier analysis assisted in estimating the OS of different subgroups, and a log-rank test helped confirm the prognostic difference. As revealed, the group with a low risk saw a smaller number of mortalities relative to the group with a high risk in each clinical subgroup (Fig. 4c), which confirmed the robustness of our signature.

### External validation of the five-gene prognostic signature in the ICGC cohort

**Fig. 5:**
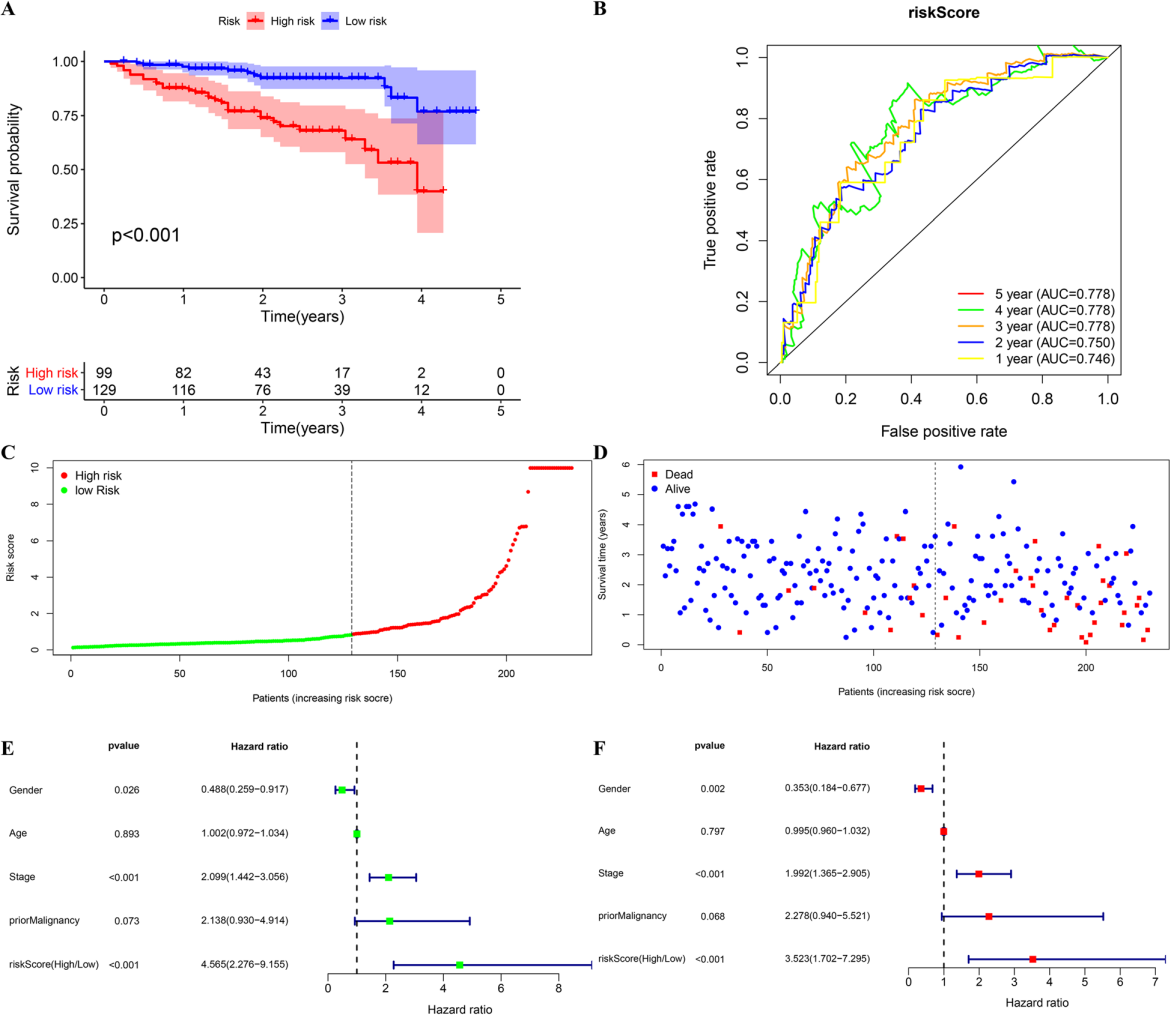
External validation of the prognostic model in ICGC cohort. **a**, **b** Kaplan-Meier survival analysis and time-dependent ROC analysis of predicting overall survival for patients in ICGC cohort used by risk score. **c**, **d** The distribution of risk score and the survival status of patients. **e**, **f** Forrest plot of the univariate and multivariate Cox regression analysis in HCC regarding OS (green represents univariate analysis, and red represents multivariate analysis)

**Fig. 6:**
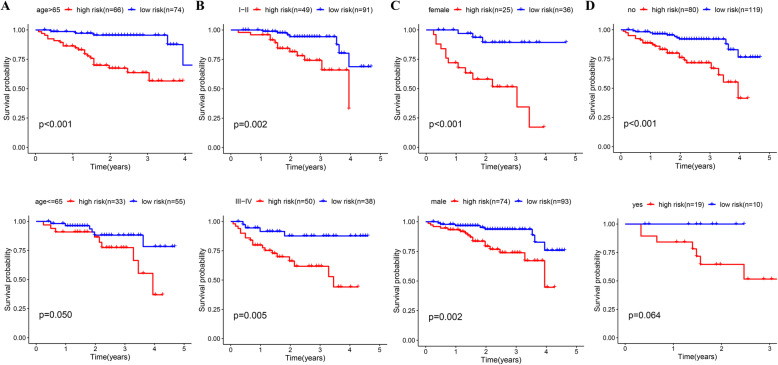
Clinical subgroup validation of the prognostic model in the ICGC cohort. **a** Age. **b** Stage. **c** Gender. **d** Prior malignancy

To further evaluate whether the prognostic model was reliable, an external dataset from the ICGC database was adopted. The risk score of the 228 patients in the ICGC cohort was taken as a criterion to divide them into a group with a high risk and a group with a low risk, which was similar to the step regarding the TCGA cohort. In line with expectations, patients in the high-risk group exhibited poorer OS (*P* < 0.001; Fig. 5a). The AUCs of the risk score in predicting 1-year, 2-year, 3-year, 4-year, and 5-year OS were 0.746, 0.750, 0.778, 0.778, and 0.778, respectively (Fig. 5b). The risk score distribution plot and survival status plot demonstrated that the risk for death increased as the risk score increased (Fig. 5c, d). In line with univariate and multivariate analyses, the risk score could help to predict the prognosis of patients (*P* < 0.001, Fig. 5e, f). It is worth mentioning that the prognostic model was also suitable for patients with different clinical features (Fig. 6a–d). These results demonstrated that successful external validation of our model was achieved.

### The prognostic model showed superior to TNM stage

**Fig. 7:**
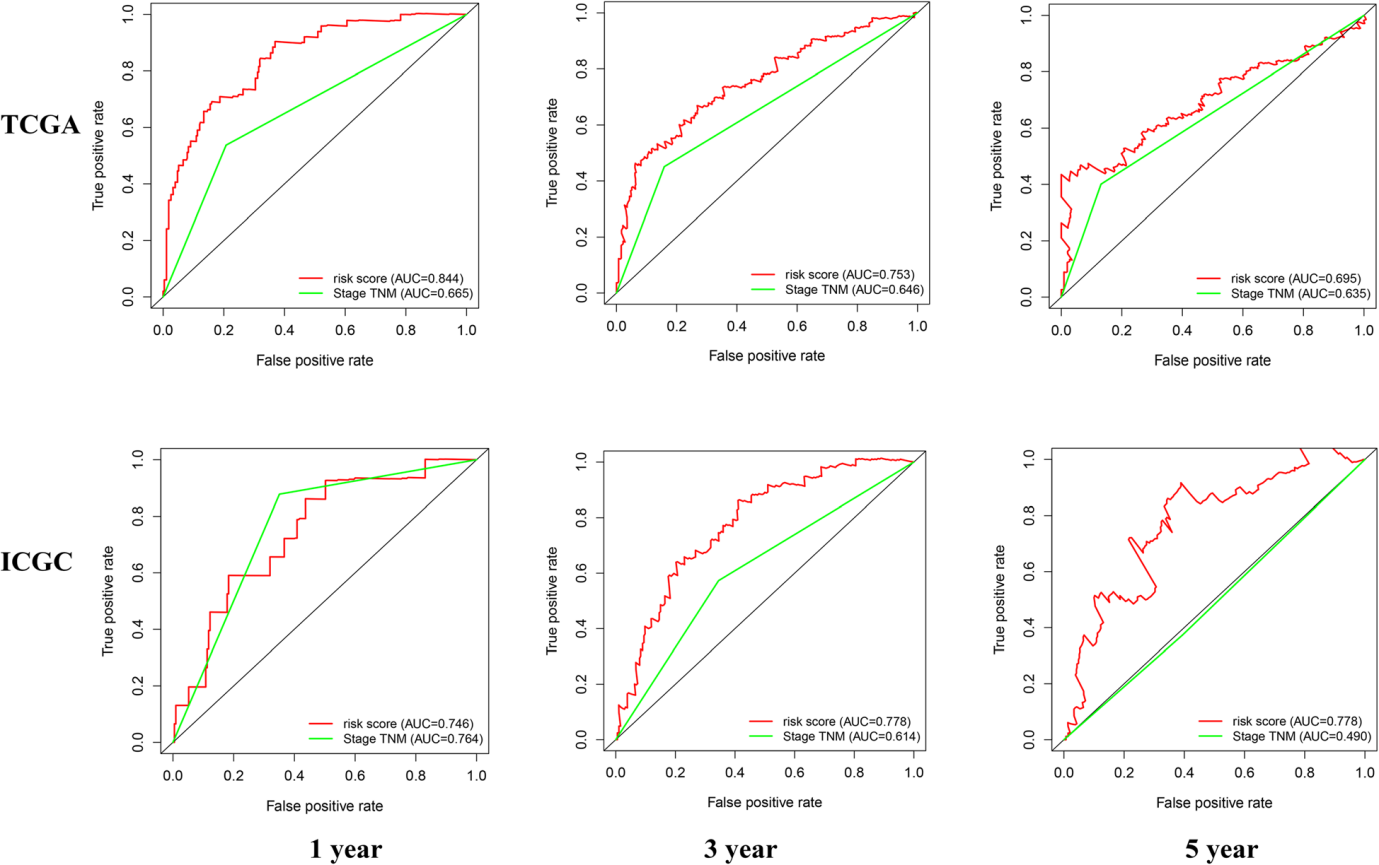
Comparison of the risk score and TNM stage’s prognostic predictive performance

The TNM stage was still the most common instrument predict the prognosis of HCC in clinical practice currently. By comparing the AUC values, we found that the risk score with better performance, especially in predicting the long-term survival of HCC (Fig. 7).

## Discussion

### HCC is a serious threat to human health

Hepatocellular carcinoma (HCC) accounts for 85–90% of primary liver cancers, is the 2nd leading cause of cancer-related death, is the 4th most common cancer worldwide, and has the 6th highest incidence [13, 14]. HCC is usually characterized by occult onset, early asymptomatic manifestation, and rapid development. Most HCC patients have progressed to the middle or advanced stage upon diagnosis [15]. Therefore, it is of great significance to prolong the survival time and improve the quality of life of patients if we can monitor the curative effect and prognosis of HCC in real time.

### The heterogeneity of HCC is a challenge

HCC is a kind of malignant tumor with high heterogeneity [16]. Even in patients in the same clinical stage, the molecular characterization of tumor cells is still very different. According to the molecular classification, different types of patients have different clinical efficacies and prognoses [17]. In recent years, as high-throughput sequencing has been developed rapidly, exploring biomarkers of immunotherapy and molecular targeted therapy based on molecular typing can accurately identify the population of patients who would benefit and predict the efficacy and prognosis of drugs, which has become a popular research topic [18].

### ARID1A mutation in HCC remains to be elucidated

In HCC, epigenetic modifications often change. ARID1A, an SWI/SNF chromatin-remodeling gene, usually is mutated in cancer and is considered to be capable of suppressing tumors [19, 20]. Sun et al. [21] revealed the context-specific function possessed by ARID1A, an SWI/SNF component, in liver cancer. Increased ARID1A facilitates tumor initiation via oxidative stress mediated by CYP450, and decreased ARID1A in established tumors strengthens metastasis because inhibitory factors present lower expression. An increasing number of studies have reported that ARID1A is associated with the OS of liver cancer patients [22, 23], but the specific mechanism is still unclear.

### ARID1A mutation may lead to the upregulation of ROS- and MYC-related genes

Our research found that HCC patients with ARID1A mutations showed substantially poor prognoses. By GSEA, we found that the genes related to reactive oxygen species (ROS) and MYC were positively correlated with ARID1A mutations. The MYC oncogene leads to many human cancers [24]. Studies that have been conducted recently regarding the expression and function of MYC have provided new insight into MYC therapy [25]. For example, drug-like molecules could inhibit bromodomain-induced MYC activation, accordingly inhibiting tumors in vivo [26]. It is also possible to suppress tumor growth through pharmacologically uncoupling bioenergetic pathways that involve glutamine or glucose metabolism from cellular biomass accumulation induced by MYC [27]. MYC can also be prevented from evolving into cancer by targeting Myc-Max dimerization or Myc-induced microRNA expression [24]. It has long been believed that unstable reactive oxygen species (ROS) promote cancer production by causing DNA damage and activating oncogenes [28, 29]. However, to date, there has been no definite answer to whether the expression of these genes is related to the prognosis of HCC.

### The prognostic signature containing 5 genes showed good performance

We extracted the genes related to reactive oxygen species (ROS) and MYC from gene expression profiles and analyzed their prognostic value for HCC. A prognostic signature consisting of 5 genes (SRXN1, LDHA, TFDP1, PPM1G, and EIF2S1) was constructed in our research. After the completion of the model construction, we carried out four levels of verification: the first was the verification of the TCGA testing cohort, namely, the internal verification; the second was the verification of the whole TCGA cohort; the third was the clinical grouping verification, namely, subgroup survival analysis; and the last was the external verification of the ICGC cohort. Through the verification of the above four dimensions, we fully affirmed the prognostic value of the prognostic signature for HCC.

### Comparison of prognostic efficacy between the five-gene prognostic signature with other previously published prognostic models

In recent years, new HCC prognostic signatures have been emerging. For example, the AUC values of the six-gene signature (SQSTM1, AHSA1, VNN2, SMG5, SRXN1, and GLS) constructed by Wenli et al. [30] were 0.761, 0.681, and 0.692, respectively, in predicting the OS of TCGA cohort in 1, 3, and 5 years, and 0.681, 0.700 and 0.684, respectively, in ICGC cohort. Another 14-gene signature (BIRC5, BUB1B, CCNB2, CDC45, DTL, GINS2, KIF23, KIF2C, MAD2L1, MCM4, OIP5, PLK4, PTTG1, and ZWINT) developed by Bo-Han Zhang et al. [31] were 0.714, 0.740, and 0.635, respectively, in predicting the OS of TCGA cohort in 1, 3, and 5 years. The AUC values for our model in predicting the OS of patients in the TCGA cohort were 0.838, 0.741, and 0.692 respectively at 1, 3, and 5 years, and 0.746, 0.778, and 0.778, respectively, in ICGC cohort. Therefore, our prognostic model is superior to the previously published prognostic models.

### The functions and pathways of the five-gene in HCC

Sulfiredoxin 1 (SRXN1) acts as a key factor regulating the antioxidant response in eukaryotic cells, can resist oxidative stress injury in cells and has antioxidant protective effects on many diseases [32]. The findings of Lv [33] demonstrated that SRXN1 modulated ROS/p65/BTG2 signaling, thereby stimulating HCC tumorigenesis and metastasis. Lactate dehydrogenase A (LDHA) serves as an important metabolic enzyme that is a member of the family of 2-hydroxy acid oxidoreductases and remarkably affects the anaerobic metabolism of cells [34]. Liu [35] reported that gankyrin upregulated LDHA expression, thereby increasing the consumption of glucose and glutamine and the production of lactate and glutamate in HCC, which might promote c-Myc-mediated tumorigenicity, metastasis, and drug resistance. The DP-1 gene (TFDP1) acts as a heterodimerization partner for E2F family members of transcription factors, and E2F/DP-1 regulates the expression of different cellular promoters, especially gene products that participate in the cell cycle [36]. TFDP1 has been identified as a c-Myc-targeted gene by D Hunecke [37], which may promote hepatocyte transformation by changing cell cycle control, thus promoting the carcinogenic activity of c-Myc. Kohichiroh Yasui [36] also found that elevated TFDP1 expression may significantly affect HCC progression, as it promoted tumor cell growth. PPM1G serves as a nuclear-localized serine/threonine phosphatase that regulates chromatin remodeling, mRNA splicing, and DNA damage [38]. Khoronenkova [39] found that the ATM-dependent protein phosphatase PPM1G dephosphorylated the ubiquitinase USP7 after ionizing radiation, resulting in the downregulation of USP7 and increased genomic instability, but its specific function has not been described previously in HCC. EIF2S1 promoted tumorigenesis by activating autophagy and enhancing tumor formation, enabling tumor cells to survive in a hypoxic and a low glucose microenvironment, making it an attractive target in Myc-driven cancer [40]. However, the function of EIF2S1 in HCC has not yet been reported.

### Future prospects

This study identified and validated a prognostic model related to ARID1A mutations for the first time, which may be a reference to better understand the pathogenesis of HCC. In addition, the method remarkably lowers the sequencing costs, ensuring more routine and cost-effective application of specific gene-based targeted sequencing, but for HCC patients who are mostly diagnosed by imaging modalities and treated with nonsurgical methods, the value of this model may be limited because our model needs to quantify the expression levels of eight specific genes in resected specimens [41]. As a retrospective study, we should acknowledge certain limitations to the study. It is necessary to conduct prospective and randomized controlled clinical studies that cover multiple centers and large sample sizes in the future. In addition, experimental analysis, including using a cell model and HCC tissues from the clinic, to validate the prediction is urgently needed.

## Conclusion

Our research proposed a novel and robust approach for the prognostic risk classification of HCC patients, and this approach may provide new insights to improve the treatment strategy of HCC.

## Supplementary Information

**Additional file 1: Supplement material 1** The workflow chart of this research.

**Additional file 2: Supplement material 2** The clinico-pathological parameter of training and validation set.

**Additional file 3: Supplement material 3** AJE language editing certificate.

**Additional file 4: Supplement material 4** The detailed information of follow-up and stage for patients in the beginning of the Kaplan meier curve between 1-2 years.

**Additional file 5: Supplement material 5** The relationship between the prognostic model and ARID1A (A) The heatmap of the prognostic model for TCGA (B) Kaplan–Meier survival analysis for the prognostic model applied on different ARID1A type in TCGA (C) The heatmap of the prognostic model for ICGC (D) Kaplan–Meier survival analysis for the prognostic model applied on different ARID1A type in ICGC.

## Data Availability

The datasets analyzed for this study were obtained from The Cancer Genome Atlas (TCGA) (https://portal.gdc.cancer.gov/) and International Cancer Genome Consortium (ICGC) (https://icgc.org/)

## Abbreviations

HCC: Hepatocellular carcinoma
TCGA: The Cancer Genome Atlas
ICGC: International Cancer Genome Consortium
GSEA: Gene set enrichment analysis
ROC: Receiver operating characteristic
OS: Overall survival
