# Supplementary figures and images for: Identification and validation of a five-gene prognostic signature for hepatocellular carcinoma
Source: World J Surg Oncol. 2021 Mar 26;19:90. doi: 10.1186/s12957-021-02202-9 (PMC8004398; doi:10.1186/s12957-021-02202-9)

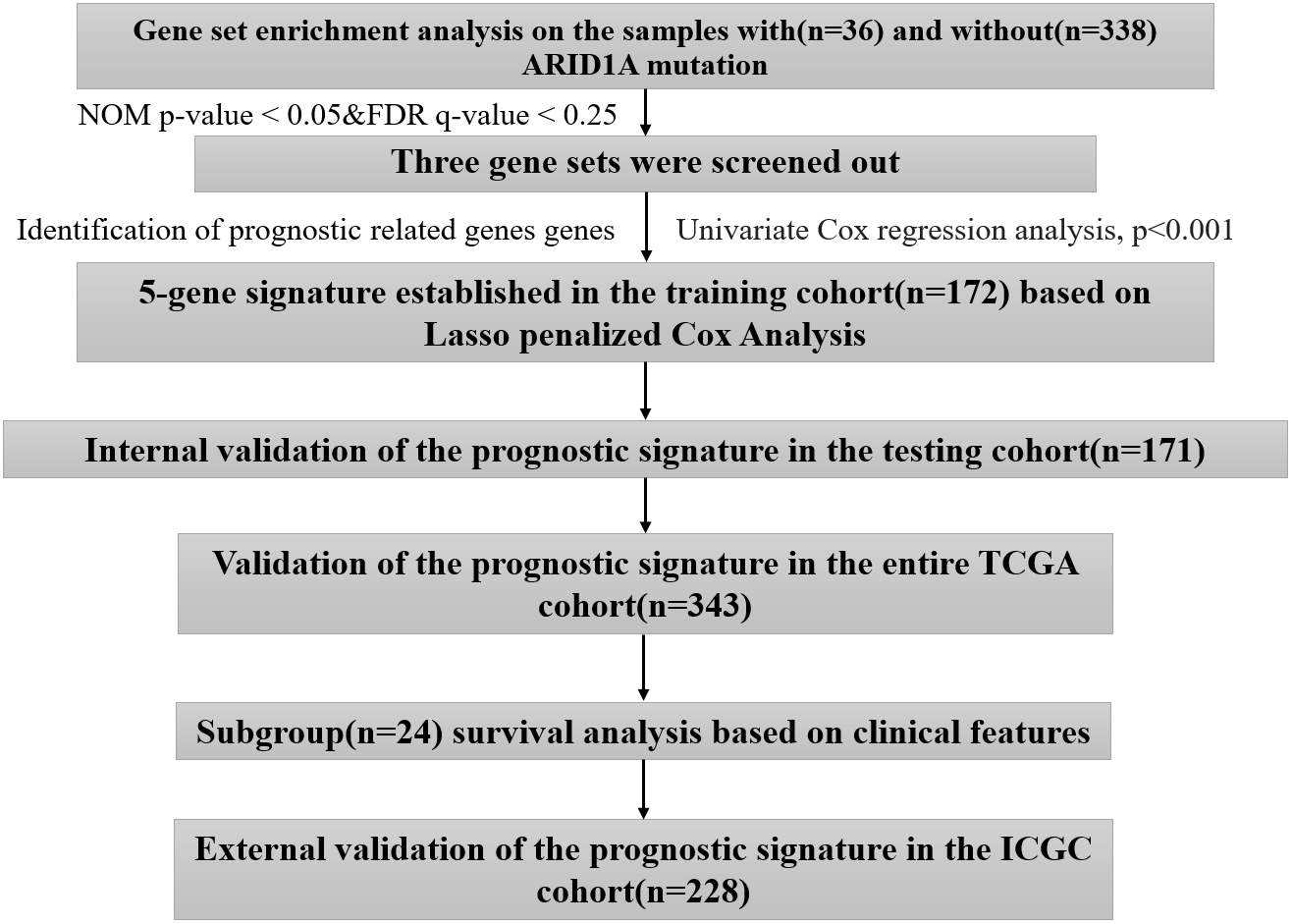

Supplement: Supplementary file 1 — Additional file 1: Supplement material 1 The workflow chart of this research. [file 12957_2021_2202_MOESM1_ESM.tif]

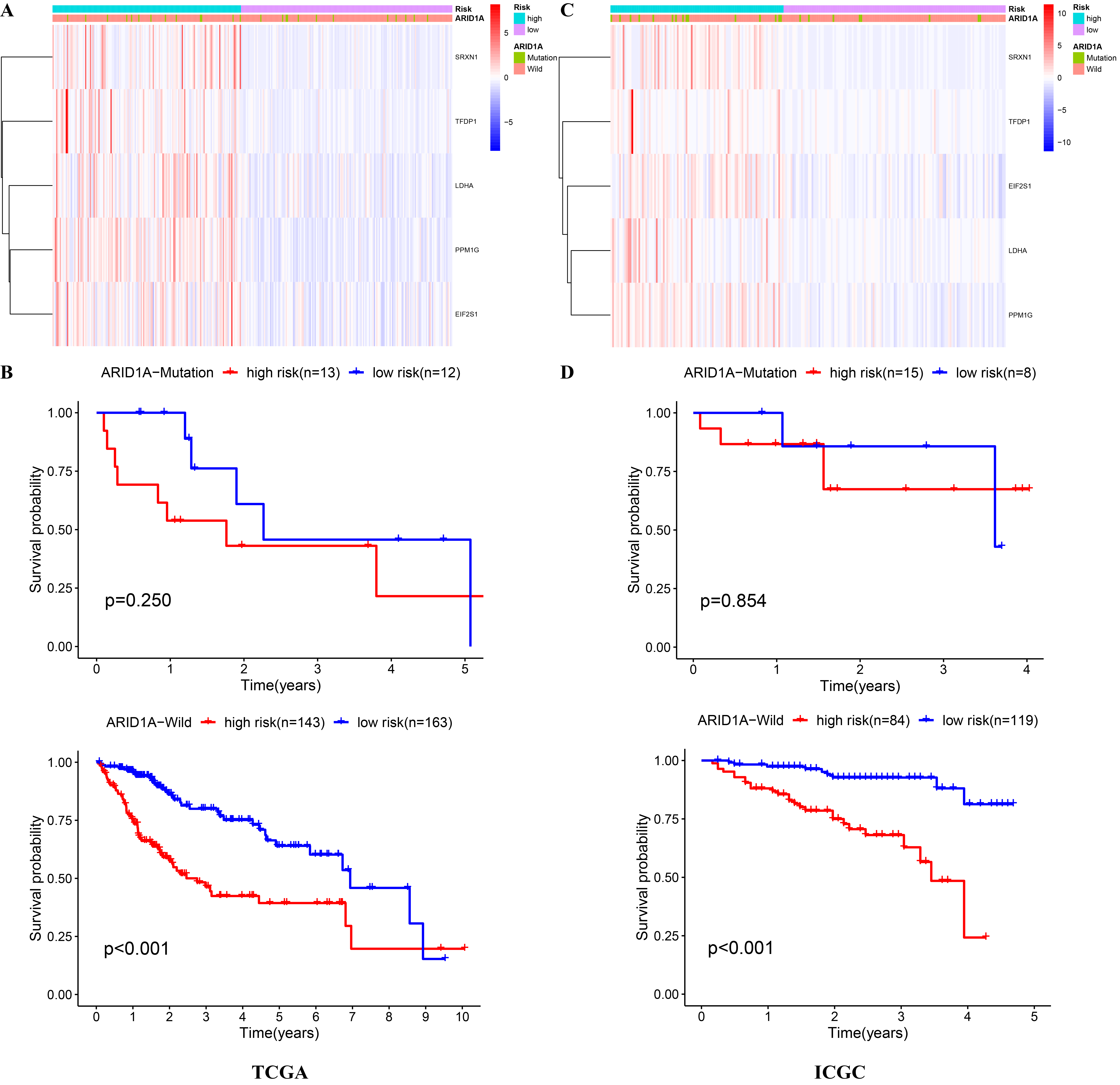

Supplement: Supplementary file 5 — Additional file 5: Supplement material 5 The relationship between the prognostic model and ARID1A (A) The heatmap of the prognostic model for TCGA (B) Kaplan–Meier survival analysis for the prognostic model applied on different ARID1A type in TCGA (C) The heatmap of the prognostic model for ICGC (D) Kaplan–Meier survival analysis for the prognostic model applied on different ARID1A type in ICGC. [file 12957_2021_2202_MOESM5_ESM.tif]
