# Supplementary material for: Identification and validation of a five-gene prognostic signature for hepatocellular carcinoma
Source: World J Surg Oncol. 2021 Mar 26;19:90. doi: 10.1186/s12957-021-02202-9 (PMC8004398; doi:10.1186/s12957-021-02202-9)
Supplement: Supplementary file 2 — Additional file 2: Supplement material 2 The clinico-pathological parameter of training and validation set. [file 12957_2021_2202_MOESM2_ESM.docx]

Supplement material 2 The clinico-pathological parameter of training and validation set

|  | Training cohort | Testing cohort | Chi-square, df | P-value |
| --- | --- | --- | --- | --- |
| Survival status | |  | 0.3679, 1 | 0.5442 |
| alive | 115 | 109 |  |  |
| dead | 57 | 62 |  |  |
| Age |  |  | 0.6792, 1 | 0.4099 |
| >65 | 60 | 67 |  |  |
| <=65 | 112 | 104 |  |  |
| Gender |  |  | 0.5347, 1 | 0.4647 |
| male | 120 | 113 |  |  |
| female | 52 | 58 |  |  |
| Grade |  |  | 1.103, 2 | 0.5762 |
| G1 | 30 | 23 |  |  |
| G2 | 78 | 83 |  |  |
| G3-4 | 64 | 60 |  |  |
| Stage TNM | |  | 2.309, 2 | 0.3152 |
| I | 88 | 73 |  |  |
| II | 38 | 39 |  |  |
| III-IV | 37 | 46 |  |  |
| Vascular tumor cell type | |  | 1.294, 2 | 0.5236 |
| None | 97 | 91 |  |  |
| Micro | 41 | 44 |  |  |
| Marco | 6 | 10 |  |  |
| Prior_malignancy | |  | 0.3002, 1 | 0.5837 |
| None | 155 | 157 |  |  |
| YES | 17 | 14 |  |  |
| Cancer status | |  | 3.405, 1 | 0.065 |
| with tumor | 68 | 81 |  |  |
| tumor free | 100 | 79 |  |  |
| New tumor event after initial treatment | | | 0.1095, 1 | 0.7407 |
| None | 86 | 89 |  |  |
| YES | 79 | 76 |  |  |
| Race |  |  | 1.093, 1 | 0.2958 |
| white | 81 | 88 |  |  |
| others | 88 | 76 |  |  |
| AFP |  |  | 1.679, 1 | 0.195 |
| <=300ng/ml | 99 | 98 |  |  |
| >300ng/ml | 37 | 25 |  |  |
| BMI |  |  | 0.2125, 1 | 0.6448 |
| >25 | 80 | 73 |  |  |
| <=25 | 81 | 82 |  |  |
| ARID1A |  |  | 0.05005, 1 | 0.823 |
| mutation | 13 | 12 |  |  |
| wild | 152 | 154 |  |  |
