# Supplementary material for: Identification and validation of a five-gene prognostic signature for hepatocellular carcinoma
Source: World J Surg Oncol. 2021 Mar 26;19:90. doi: 10.1186/s12957-021-02202-9 (PMC8004398; doi:10.1186/s12957-021-02202-9)
Supplement: Supplementary file 3 — Additional file 3: Supplement material 3 AJE language editing certificate. [file 12957_2021_2202_MOESM3_ESM.pdf]

Editing Certificate

This document certifies that the manuscript

Identification and validation of a five-gene prognostic signature associated with ARID1A alteration for hepatocellular carcinoma

prepared by the authors

Huibin Yang, Junyu Huo, Xin Li

was edited for proper English language, grammar, punctuation, spelling, and overall style by one or more of the highly qualified native English speaking editors at AJE.

This certificate was issued on **February 10, 2021** and may be verified on the AJE website using the verification code **3287-5162-DEE8-6F0C-2135**.

Neither the research content nor the authors' intentions were altered in any way during the editing process. Documents receiving this certification should be English-ready for publication; however, the author has the ability to accept or reject our suggestions and changes. To verify the final AJE edited version, please visit our verification page at aje.com/certificate. If you have any questions or concerns about this edited document, please contact AJE at support@aje.com.

AJE provides a range of editing, translation, and manuscript services for researchers and publishers around the world. For more information about our company, services, and partner discounts, please visit aje.com.
